# Cancer cachexia onset and survival outcomes in metastatic colorectal cancer: Comparative assessment of the asian working group for cachexia and the European palliative care research collaborative criteria, and utility of modified glasgow prognostic score

**DOI:** 10.1007/s00384-025-04962-2

**Published:** 2025-07-25

**Authors:** Hironori Fujii, Misato Kani, Daichi Watanabe, Yuto Miura, Wakana Chikaishi, Jesse Yu Tajima, Akitaka Makiyama, Yunami Yamada, Koichi Ohata, Chiemi Hirose, Hirotoshi Iihara, Ryo Kobayashi, Nobuhisa Matsuhashi, Akio Suzuki

**Affiliations:** 1 Department of Pharmacy, Gifu University Hospital, Gifu, Japan; 2 Laboratory of Advanced Medical Pharmacy, Gifu Pharmaceutical University, Gifu, Japan; 3 Innovative and Clinical Research Promotion Center, Gifu University Hospital, Gifu, Japan; 4 Department of Surgical Oncology, Gifu University Graduate School of Medicine, Gifu, Japan; 5 Cancer Center, Gifu University Hospital, Gifu, Japan; 6 Patient Safety Division, Gifu University Hospital, Gifu, Japan; 7 Laboratory of Pharmacy Practice and Social Science, Gifu Pharmaceutical University, Gifu, Japan

**Keywords:** Cancer cachexia, Metastatic colorectal cancer, AWGC criteria, EPCRC criteria, Modified Glasgow Prognostic Score, Prognostic value, Time-dependent analysis

## Abstract

**Objective:**

Cachexia substantially affects the prognosis of patients with advanced cancer. While both the Asian Working Group for Cachexia (AWGC) and European Palliative Care Research Collaborative (EPCRC) criteria are widely used for diagnosis, their comparative effectiveness in diagnostic timing and prognostic value remain understudied.

**Methods:**

This retrospective study included patients with metastatic colorectal cancer (mCRC) who received first-line chemotherapy between 2013 and 2023. Cachexia was assessed using three distinct criteria: AWGC criteria, defined as either weight loss > 2%, or body mass index (BMI) < 21 kg/m^2^ accompanied by at least one of the following: anorexia or elevated C-reactive protein level; EPCRC criteria, requiring weight loss > 5% (or weight loss > 2% if BMI is < 20); and Modified Glasgow Prognostic Score (mGPS). Analyses were performed using cumulative incidence and survival with a time-dependent Cox regression model.

**Results:**

We enrolled 313 patients with metastatic CRC. The 1-year cumulative incidence of cachexia showed marked variation across the different diagnostic criteria. Using the AWGC criteria, the incidence rate was 69%, whereas the EPCRC criteria identified 44% of the cases. The mGPS evaluation revealed incidence rates of 73% and 39% for scores of 1 and 2, respectively. Both AWGC- and EPCRC-defined cachexia correlated with significantly shorter overall survival (AWGC: hazard ratio (HR) = 2.41, *P* < 0.001; EPCRC: HR = 2.02, *P* < 0.001). Similarly, the mGPS scores indicated a poor prognosis.

**Conclusion:**

The AWGC criteria identified a higher incidence of cachexia earlier in the disease course compared to the EPCRC criteria and showed a stronger association with overall survival. The mGPS shows promise as an alternative diagnostic tool to traditional weight-based assessments. These findings suggest new opportunities for early diagnosis of cachexia and intervention strategies in patients with mCRC.

## Introduction

Cancer cachexia is a complex nutritional syndrome characterized by weight loss and anorexia, particularly loss of muscle mass, which diminishes the quality of life (QOL) and worsens the prognosis of patients with cancer [1–3]. Although weight loss is essential for diagnosing cancer cachexia, there is no consensus on the amount of weight a patient must lose to warrant a diagnosis. The traditional international consensus by Fearon et al. defined cachexia as weight loss greater than 5% over a 6-month period (or > 2% in individuals with a body mass index [BMI] < 20 or sarcopenia) [4]. More recently, the Asian Working Group for Cachexia (AWGC) has established criteria, defining cachexia as a weight loss > 2% over 3–6 months plus at least one of anorexia, reduced grip strength (< 28 kg for men and < 18 kg for women), or C-reactive protein (CRP) level of > 0.5 mg/dL [5]. However, it remains unclear whether these criteria or those of the EPCRC (European Palliative Care Research Collaborative) are more appropriate for Japanese patients, particularly those undergoing chemotherapy.

Treatment of cancer cachexia requires a multimodal approach that combines pharmacotherapy, exercise, and nutritional intervention [6]. In Japan, anamorelin has been approved for the treatment of cachexia, offering potential improvements in lean body mass and appetite [7–10]. However, early intervention is hindered by uncertainty regarding the timing of cachexia onset and insufficient establishment of non-weight-based evaluation markers.

A retrospective study of patients with colorectal cancer reported that approximately 40% developed cancer cachexia within 0–12 weeks of initiation of first-line chemotherapy [11]. However, this study only considered weight loss (> 5% over 6 months or > 2% with a BMI < 20) without evaluating anorexia. Recent reports indicate that the survival periods for patients with unresectable advanced colorectal cancer now exceed 30 months [12–14]. Therefore, identifying the timing of the onset of cachexia during long-term chemotherapy is crucial for early multimodal interventions.

Regular weight measurements for all patients receiving chemotherapy can be challenging, necessitating alternative indicators. The modified Glasgow Prognostic Score (mGPS), which is scored between 0 and 2 based on CRP and albumin (ALB) levels and reflects systemic inflammation and nutritional status, is one potential candidate [15]. Higher mGPS scores are correlated with cancer progression, systemic inflammatory response, and poor prognosis [16]. It is also useful as an indicator to guide nutritional management and treatment planning in patients with cancer cachexia and chronic diseases [17, 18]. In addition, mGPS has been reported as a predictive marker for cachexia [4, 5]. However, the relationship between mGPS and timing of cachexia onset remains unclear, and studies examining the correlation between weight loss and mGPS are limited.

Therefore, there is an urgent need to identify the appropriate diagnostic criteria for cancer cachexia in Japanese patients undergoing chemotherapy. The aim of this study was to clarify the timing of cachexia onset in patients with metastatic colorectal cancer (mCRC) by comparing the EPCRC and AWGC criteria. Furthermore, we assessed the utility of mGPS as an alternative screening marker and prognostic indicator when routine weight monitoring is not feasible in clinical practice.

## Methods

### Setting and participants

This retrospective observational study used electronic medical records from our institution. The study population comprised all consecutive patients with mCRC who started first-line cancer chemotherapy between January 1, 2013, and December 31, 2023. All 313 patients included in this study met the predefined inclusion criteria (confirmed diagnosis of metastatic colorectal cancer, initiation of first-line chemotherapy during the study period, available baseline demographic and clinical data, and adequate follow-up data for survival analysis), and no patients were excluded from the final analysis once they met these criteria. This study was approved by the Ethics Committee of Gifu University Graduate School of Medicine (approval number 2023–289) and was conducted in accordance with the Japanese government guidelines for human research. The requirement for informed consent was waived owing to the retrospective nature of the study.

### Cancer cachexia assessment

The EPCRC criteria defines cachexia as a weight loss > 5% from baseline or > 2% in patients with a BMI < 20 kg/m^2^ [4]. Owing to the retrospective design of this study, the sarcopenia assessment included in the EPCRC criteria could not be performed due to the lack of consistent imaging data suitable for skeletal muscle mass evaluation. The AWGC criteria defined cachexia as either (1) BMI < 21 kg/m^2^, or (2) weight loss > 2% from baseline over 3–6 months accompanied by at least one of the following: anorexia or CRP > 0.5 mg/dL [5]. While the AWGC criteria also include reduced grip strength, this parameter could not be evaluated retrospectively due to lack of available data.

The mGPS, an indicator of systemic inflammation and nutritional status in patients with cancer, was evaluated using the following criteria: score 0 (CRP ≤ 0.5 mg/dL and ALB ≥ 3.5 mg/dL), score 1 (CRP > 0.5 mg/dL and ALB ≥ 3.5 mg/dL), and score 2 (CRP > 0.5 mg/dL and ALB < 3.5 mg/dL) [16]. The CRP cut-off of > 0.5 mg/dL was used based on previous clinical studies in Japanese populations [19], where this threshold in combination with low albumin (< 3.5 mg/dL) has been shown to have statistically significant prognostic performance for mGPS score 2. This differs from the > 10 mg/L (equivalent to 1.0 mg/dL) threshold used in some Western studies [15]. Both mGPS 1 and 2 were analyzed as potential cachexia indicators when weight measurements were impractical.

### Anorexia assessment

Anorexia was assessed based on medical chart documentation of appetite loss. Symptomatic anorexia was defined as Grade 1 or higher according to CTCAE v4.0 [20]. All patients had available data for anorexia assessment. The timing of anorexia onset was approximated based on documentation of symptoms in clinical records.

### Statistical analysis

Patient characteristics are summarized as medians (interquartile range [IQR], 25th–75th percentiles) for continuous variables and as counts (percentages) for categorical variables. The cumulative incidence of cancer cachexia defined by the EPCRC, AWGC, mGPS 1, and mGPS 2 was estimated using a cumulative incidence function. Because death precludes the diagnosis of cachexia, it was treated as a competing risk to avoid overestimation of the incidence that can occur with standard survival methods. This analysis was implemented using the R package cmprsk. (R Foundation for Statistical Computing, Vienna, Austria).

For patients who met both EPCRC and AWGC criteria during the observation period, the time (in days) from the initiation of first-line chemotherapy to the point each criterion was met was calculated. Spearman's rank correlation coefficient and its 95% confidence intervals (CIs) were determined to assess the relationship between the onset times of cachexia as defined by these two criteria.

Overall survival (OS) was assessed using the Simon and Makuch-modified Kaplan–Meier method. Time-dependent Cox proportional hazards regression analyses were performed to evaluate the prognostic impact of cachexia as defined by each criterion (EPCRC, AWGC, mGPS 1, and mGPS 2). These models accounted for cachexia status as a time-varying covariate and were adjusted for potential confounders (age, sex, neutrophil-to-lymphocyte ratio [NLR], and baseline mGPS). Given the potential prognostic relevance of systemic inflammation, baseline mGPS was incorporated as a covariate in all multivariable time-dependent Cox models assessing the impact of cachexia (AWGC and EPCRC) on overall survival. Hazard ratios (HRs) and 95% CIs were calculated. Additionally, to evaluate the discriminative performance of each cachexia definition, concordance index (C-index) values were calculated from time-dependent Cox regression models.

Statistical significance was defined as a two-sided *P*-value < 0.05. All analyses were performed using R version 4.2.2 (R Foundation for Statistical Computing).

## Results

### Patient characteristics

This study enrolled 313 patients with mCRC who received first-line treatment, including oxaliplatin (Table 1). The median age was 68 years (range: 59–74 years) and males comprised 56.2% (176 patients) of the cohort. The median body weight was 56.0 kg (49.4–64.0 kg), and the median BMI was 21.7 (19.2–24.4). Laboratory values showed a median serum ALB level of 4.0 g/dL (3.6–4.2), a median CRP level of 0.2 mg/dL (0.1–0.8), and a median NLR of 2.3 (1.7–3.5). The distribution of mGPS scores was as follows: 79.1% (235 patients) for score 0, 10.1% (30 patients) for score 1, and 10.8% (32 patients) for score 2.

**Table 1:** Patient characteristics

Variable	N	Median (IQR)/Count (%)	Unknown (n)
Age (y)	311	68 (59, 74)	2
Male sex	313	176 (56.2%)	0
Weight (kg)	313	56.0 (49.4, 64.0)	0
BMI	308	21.7 (19.2, 24.5)	5
Albumin (mg/dL)	297	4.0 (3.6, 4.2)	16
Total Protein (mg/dL)	290	6.8 (6.4, 7.2)	23
Aspartate Aminotransferase (U/L)	297	20.0 (17.0, 29.0)	16
Alanine Aminotransferase (U/L)	297	16.0 (11.0, 24.0)	16
Total Bilirubin (mg/dL)	295	0.6 (0.5, 0.8)	18
White Blood Cell Count (/μL)	298	5,620 (4,750, 6,870)	15
Neutrophil Count (/μL)	297	3,440 (2,567, 4,680)	16
Lymphocyte Count (/μL)	298	1,450 (1,158, 1,917)	15
C-Reactive Protein (mg/dL)	299	0.2 (0.1, 0.8)	14
Neutrophil-to-Lymphocyte Ratio	296	2.3 (1.7, 3.6)	17
Modified Glasgow Prognostic Score	297	0: 235 (79.1%)	16
		1: 30 (10.1%)	
		2: 32 (10.8%)	
Carcinoembryonic Antigen (ng/mL)	302	14.9 (4.3, 74.7)	11

*IQR* interquartile range, *BMI* body mass index

### Cumulative incidence of cachexia by different criteria

**Fig. 1:**
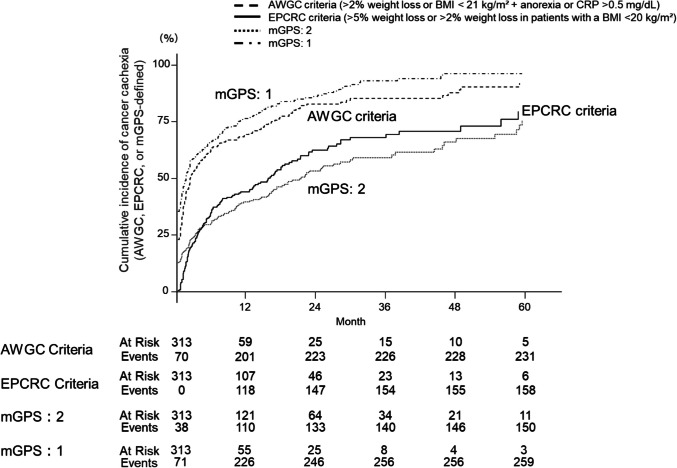
Cumulative incidence of cancer cachexia (EPCRC and AWGC criteria) and mGPS after chemotherapy initiation in patients with metastatic colorectal cancer. Abbreviations: EPCRC, European Palliative Care Research Collaborative; AWGC, Asian Working Group for Cachexia

The incidence of cachexia was consistently higher when assessed using the AWGC criteria and an mGPS score of 1, suggesting that these measures may be more sensitive for early detection (Fig. 1). The one-year cumulative incidence of cachexia was 69% (95% CI: 63–74%) using the AWGC criteria and 44% (95% CI: 37–50%) using the EPCRC criteria. These cumulative incidences were calculated from the initiation of first-line chemotherapy. For mGPS, the cumulative incidence at 1 year was 73% (95% CI: 67–78%) for score 1 and 39% (95% CI: 33–45%) for score 2. Among 313 patients, the cumulative incidence of weight loss over time showed distinct patterns: at one year, > 2% weight loss was observed in approximately 188 patients (60%; 95% CI: 53–65%), and > 5% weight loss in approximately 116 patients (37%; 95% CI: 31–43%). These numbers were estimated using time-to-event analysis and reflect survival-adjusted incidences rather than simple proportions. Among patients who met both the EPCRC and AWGC criteria, a significant positive correlation was observed between the time to meeting EPCRC criteria and the time to meeting AWGC criteria (Spearman's ρ = 0.535; 95% CI: 0.387–0.664; *P* < 0.001).

### Association between cachexia onset and survival

**Fig. 2:**
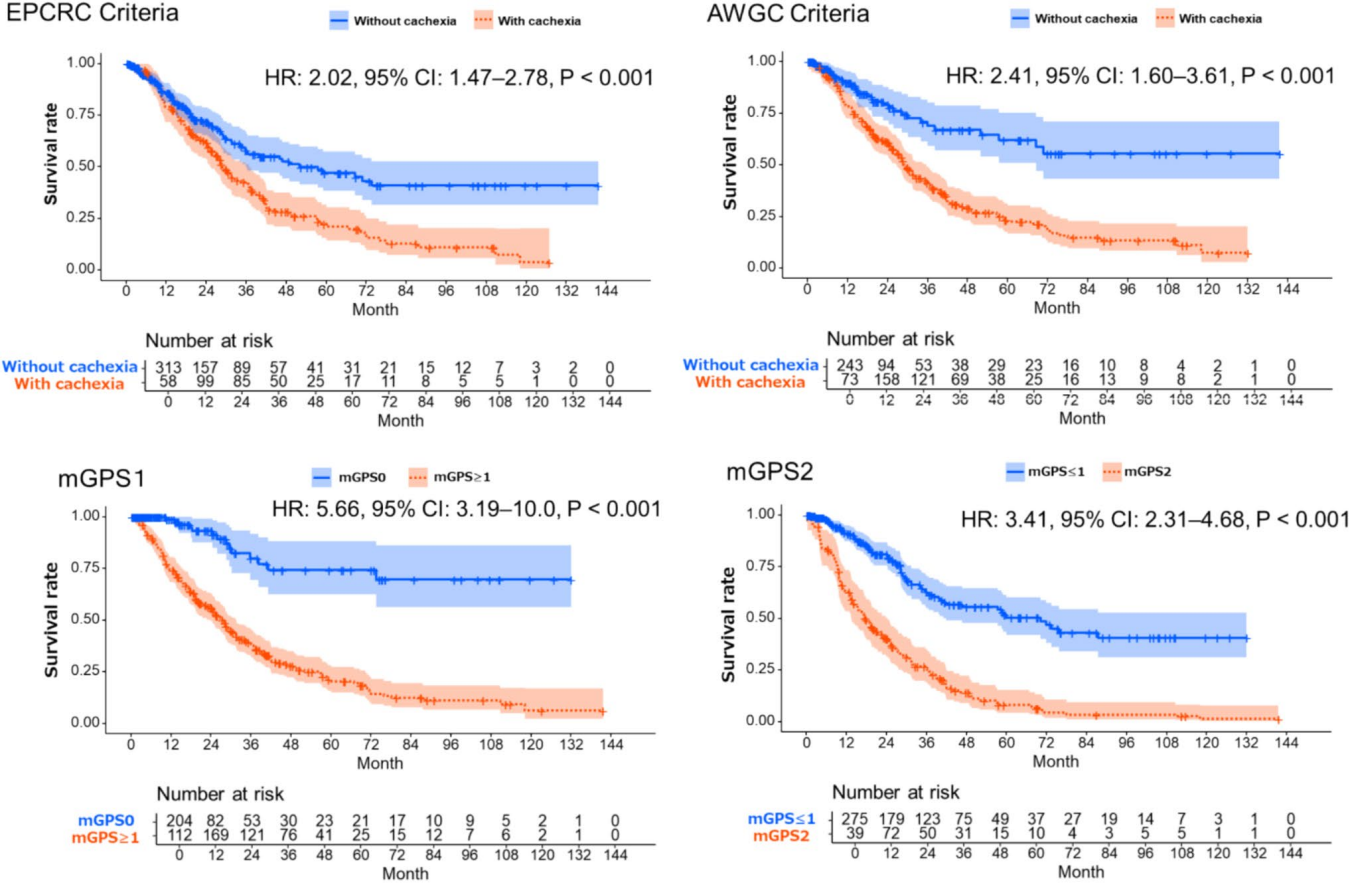
Simon and Makuch’s modified Kaplan–Meier curves for overall survival based on cancer cachexia (EPCRC and AWGC criteria) and mGPS in patients with metastatic colorectal cancer. Abbreviations: EPCRC, European Palliative Care Research Collaborative; AWGC, Asian Working Group for Cachexia

A survival analysis using Simon and Makuch’s modified Kaplan–Meier curves with OS as the outcome and cachexia onset (by both criteria) and mGPS 1 or 2 as time-dependent exposure factors was performed (unadjusted curves are presented in Fig. 2). The median follow-up time for the entire cohort was 52.3 months (95% CI: 44.3–66). The median overall survival was 35.7 months (95% CI: 30.3–41.8) for the entire cohort. Time-dependent Cox regression analysis adjusted for age, sex, and NLR revealed significant associations between all exposure factors and shortened survival (Table 2). Specifically, patients who developed cachexia according to AWGC criteria showed an HR of 2.41 (95% CI: 1.60–3.61, *P* < 0.001), while those meeting EPCRC criteria showed an HR of 2.02 (95% CI: 1.47–2.78, *P* < 0.001). Similarly, patients with mGPS scores of 1 and 2 demonstrated significantly shortened survival, with HRs of 5.66 (95% CI: 3.19–10.0, *P* < 0.001) and 3.41 (95% CI: 2.31–4.68, *P* < 0.001), respectively (Fig. 2). We assessed the discriminative performance of each cachexia definition using C-index values derived from time-dependent Cox regression models. In univariate analyses, the C-index was 0.559 for EPCRC, 0.584 for AWGC, 0.613 for mGPS1, and 0.656 for mGPS2.

**Table 2:** Time-dependent Cox proportional hazards regression to assess the prognostic impact of cancer cachexia according to EPCRC, AWGC, and mGPS

Factors	Hazard Ratio (HR)	95% Confidence Interval (CI)	*P*-value
AWGC Criteria	2.41	1.60–3.61	< 0.001
EPCRC Criteria	2.02	1.47–2.78	< 0.001
mGPS = 1	5.66	3.19–10.0	< 0.001
mGPS = 2	3.41	2.31–4.68	< 0.001

All models were adjusted for age, sex, neutrophil-to-lymphocyte ratio (NLR), and baseline mGPS

## Discussion

In this study, we evaluated the timing of the onset of cancer cachexia and its effect on the survival of patients with mCRC using different diagnostic criteria (EPCRC, AWGC, and mGPS). Our findings revealed that cachexia, as defined by the AWGC criteria, developed in 69% of patients within 1 year, suggesting that these criteria identify a larger proportion of patients with cachexia earlier in the course of disease compared with the EPCRC criteria (44% at 1 year). The AWGC criteria identified cachexia in a larger proportion of patients and at an earlier stage of disease progression, as evidenced by the cumulative incidence curves, which may enable earlier clinical intervention. Furthermore, cachexia diagnosis based on the AWGC criteria showed a higher hazard ratio and a significant association with shortened survival compared with the EPCRC criteria, highlighting the importance of AWGC-based monitoring.

Our results demonstrate a 44% cumulative cachexia incidence at 12 months using the EPCRC criteria, which aligns with Shibata et al.’s reported incidence of 40% [11], thereby supporting the generalizability of our study results. To the best of our knowledge, this is the first retrospective study to examine the cumulative incidence of cachexia using the AWGC criteria in patients with mCRC, representing a significant contribution to the field. Wang et al. demonstrated that the AWGC criteria is an independent prognostic factor [21], and our study confirms the significant association between the AWGC criteria and survival outcomes, suggesting its suitability for use in Asian populations.

The higher cumulative incidence of cachexia using the AWGC criteria compared with the EPCRC criteria, coupled with its stronger association with OS, warrants attention. Katsushima et al.’s study of 106 patients with lung cancer showed similar patterns, with the AWGC criteria identifying more cases (72.6%) than the EPCRC criteria (54.7%) [22]. While the EPCRC criteria have been widely used in Japan, including for anamorelin indication, differences in body composition and dietary habits between Western and Asian populations may necessitate race-specific diagnostic criteria. A moderate positive correlation (Spearman's ρ = 0.535, *P* < 0.001) was found between the time to onset of cachexia defined by AWGC and EPCRC criteria among patients who met both, suggesting a shared underlying progression of cachexia despite differences in diagnostic sensitivity. This correlation indicates that while both criteria capture the same pathophysiological process, the AWGC criteria demonstrate superior sensitivity for early detection. Regarding cancer-specific variations, using the EPCRC criteria, Mitsunaga et al. reported a 64% incidence of cachexia within 12 months in patients with advanced pancreatic adenocarcinoma [23], which is notably higher than the 44% observed in patients with mCRC in our study. This difference likely reflects the more aggressive nature of pancreatic cancer and its pronounced impact on nutritional status and systemic inflammation, suggesting that cachexia development patterns vary significantly across cancer types and should inform treatment strategies.

The established role of mGPS stems from its ability to quantify key pathophysiological elements of cachexia, namely systemic inflammation and nutritional status [15, 16], making it a crucial marker in assessing the underlying biological processes of cachexia rather than just weight loss as a symptom. This aligns with its recognized use in frameworks like the Global Leadership Initiative on Malnutrition (GLIM) where it serves as a criterion for systemic inflammation. Furthermore, its consistent prognostic value across various cancer types [16] and other chronic diseases such as heart failure and chronic kidney disease, and it can identify patients at risk earlier, even in the absence of significant weight loss, as highlighted in prior literature on mGPS [25].

Our investigation of the utility of mGPS aligns with previous studies showing a significant correlation with mCRC survival rates [24]. The high frequency of elevated mGPS in patients with cachexia supports its value as a prognostic indicator, which is consistent with the findings of Liying et al.’s systematic review and meta-analysis [25]. Our study indicates that both weight loss and mGPS are important prognostic factors in mCRC.

We interpreted these results by emphasizing that mGPS2 had the highest discriminative ability, followed by mGPS1 and AWGC. Although the differences were modest, C‑index values in the range of 0.61–0.66 are considered clinically meaningful in complex disease settings such as metastatic colorectal cancer, as C‑index values of 0.6–0.7 are widely accepted as indicative of useful prognostic discrimination in oncology research [26, 27]. These results support the prognostic relevance of mGPS and AWGC in cachexia assessment.

In the clinical setting, especially during outpatient chemotherapy, regular weight monitoring can be challenging because of time constraints, varying visit intervals, and patient conditions. The mGPS, which can be calculated from routine laboratory tests, offers a practical and objective tool for screening patients at risk of cachexia and initiating timely interventions. Our finding that both mGPS scores of 1 and 2 significantly impacted OS suggests that interventions should begin at a score of 1 rather than waiting for a score of 2. While we observed higher incidences of cachexia with AWGC criteria (which heavily relies on weight loss) and mGPS score 1, the retrospective design limits our ability to definitively determine the temporal precedence or relative importance between weight loss and inflammation (mGPS). Further prospective studies are needed to clarify their precise interplay and independent contributions to prognosis.

Successful treatment of cachexia requires a multimodal approach that combines pharmacotherapy (e.g., anamorelin), nutritional support, and exercise therapy [28–30]. Our results emphasize the potential benefits of early detection and intervention, highlighting the importance of continuous follow-up and comprehensive support from healthcare teams.

This study had several limitations. First, given its retrospective design, selection bias cannot be ruled out. A significant limitation of this retrospective study is the unavailability of certain data points crucial for complete cachexia assessment. Specifically, handgrip strength, a component of the AWGC criteria, was not routinely measured, and consistent imaging data for skeletal muscle mass assessment, essential for the EPCRC criteria, were lacking. These omissions may have influenced the overall prevalence and characteristics of cachexia identified by each criterion. Second, the intervals between weight measurements were not standardized, which may have affected the accuracy of the timing of the onset of cachexia. Third, the retrospective assessment of anorexia relied on clinical documentation, which may not have captured all symptomatic cases owing to underreporting. Despite these limitations, our study provides valuable real-world evidence comparing different cachexia criteria in clinical practice, where complete data collection is often challenging. Prospective validation and more comprehensive analyses, including the assessment of sarcopenia, are needed. Future interventional studies are required to evaluate the specific effects of treatments on cancer cachexia.

## Conclusion

In conclusion, the AWGC criteria identified a higher incidence of cancer cachexia earlier and demonstrated stronger associations with OS. These findings support integration of the AWGC criteria and mGPS as practical screening tools into routine clinical practice for early cachexia detection in Asian populations. Future research should focus on prospective validation and strategies to improve patient outcome.

## Data Availability

No datasets were generated or analysed during the current study.
